# Relationship of dysglycemia to acute myocardial infarct size and cardiovascular outcome as determined by cardiovascular magnetic resonance

**DOI:** 10.1186/1532-429X-12-61

**Published:** 2010-11-02

**Authors:** Adam N Mather, Andrew Crean, Nik Abidin, Gillian Worthy, Stephen G Ball, Sven Plein, John P Greenwood

**Affiliations:** 1 Division of Cardiovascular and Neuronal Remodelling, University of Leeds, Leeds, UK; 2 Peter Munk Cardiac Center, Toronto General Hospital, Ontario, Canada; 3 Salford Royal University Teaching Hospital, Manchester, UK; 4 Clinical Trials Research Unit, University of Leeds, Leeds, UK

## Abstract

**Background:**

Improved outcomes for normoglycemic patients suffering acute myocardial infarction (AMI) over the last decade have not been matched by similar improvements in mortality for diabetic patients despite similar levels of baseline risk and appropriate medical therapy. Two of the major determinants of poor outcome following AMI are infarct size and left ventricular (LV) dysfunction.

**Methods:**

Ninety-three patients with first AMI were studied. 22 patients had diabetes mellitus (DM) based on prior history or admission blood glucose ≥11.1 mmol/l. 13 patients had dysglycemia (admission blood glucose ≥7.8 mmol/l but <11.1 mmol/l) and 58 patients had normoglycemia (admission blood glucose <7.8 mmol/l). Patients underwent cardiac magnetic resonance (CMR) imaging at index presentation and median follow-up of 11 months. Cine imaging assessed LV function and late gadolinium contrast-enhanced imaging was used to quantify infarct size. Clinical outcome data were collected at 18 months median follow-up.

**Results:**

Patients with dysglycemia and DM had larger infarct sizes by CMR than normoglycemic patients; at baseline percentage LV scar (mean (SD)) was 23.0% (10.9), 25.6% (12.9) and 15.8% (10.3) respectively (p = 0.001), and at 11 months percentage LV scar was 17.6% (8.9), 19.1% (9.6) and 12.4% (7.8) (p = 0.017). Patients with dysglycemia and DM also had lower event-free survival at 18 months (p = 0.005).

**Conclusions:**

Patients with dysglycemia or diabetes mellitus sustain larger infarct sizes than normoglycemic patients, as determined by CMR. This may, in part, account for their adverse prognosis following AMI.

## Introduction

The presence of elevated blood glucose levels, diabetes mellitus (DM), or both, contributes to more than 3 million cardiovascular deaths worldwide each year [1]. Patients with DM have higher mortality after acute myocardial infarction (AMI) than non-diabetic patients [2]. Moreover, patients with abnormal glucose tolerance at the time of AMI have a high subsequent cardiovascular event rate, comparable to patients with a previous history of DM [3,4].

Alegria and colleagues have highlighted many potential reasons why patients with impaired glucose tolerance and DM have a high cardiovascular event rate [5]. These include a greater degree of coronary artery atheroma [6], intrinsic systolic and diastolic left ventricular (LV) dysfunction [7,8], impaired recruitment of collateral vessels [9], a higher rate of reinfarction [10], impaired ischemic preconditioning [11], reduced myocardial perfusion with thrombolysis and primary angioplasty [12] and underuse of evidence-based therapies [13].

Two of the major determinants of poor outcome following AMI are size of infarction and subsequent LV dysfunction [14]. Cardiovascular magnetic resonance (CMR) can be used to accurately image the extent of both acute and chronic myocardial infarction using the technique of late gadolinium enhancement (LGE) [15]. The high spatial resolution of LGE-CMR has been shown to detect subendocardial infarction better than single photon emission computed tomography (SPECT) [16]. CMR also provides accurate and reproducible assessment of cardiac function such that it is now the reference standard for determining LV mass and function [17].

The aims of this study were: to determine whether there is a significant difference in infarct size and LV remodeling following AMI between non-diabetic subjects and those with dysglycemia or established DM, and to assess the impact of glycemic status on prognosis.

## Materials and methods

### Subjects

Ninety-seven patients with first presentation AMI were prospectively recruited into the study between January 2004 and December 2005. Four patients were unable to complete CMR imaging due to claustrophobia. Therefore, the study population consisted of ninety-three patients (79 men and 14 women, mean age 58 years, range 30-78 years). The study was approved by the institutional Research Ethics Committee and complied with the Declaration of Helsinki; written informed consent was obtained from all patients. Patients were recruited consecutively if they met the inclusion criteria of first presentation AMI and were willing to give consent. The diagnosis of AMI was based on previously published consensus criteria [18]. Patients were excluded from the study if they had a previous history of acute coronary syndrome, coronary artery revascularization (percutaneous coronary intervention (PCI) or coronary artery bypass graft surgery) or any contraindication to MR imaging. Further exclusion criteria included age <18 or >79 years and severe renal failure (defined as estimated glomerular filtration rate <30 ml/min/1.73 m^2^).

All patients had plasma samples for random glucose measured by the glucose oxidase/Trinders method (Bayer ADVIA 1650/2400 systems, Bayer plc, Newbury, UK). Samples were taken at the time of hospital admission and a glucose level ≥11.1 mmol/l was considered as a new diagnosis of DM, in accordance with the guidelines of the American Diabetes Association [19]. Patients with a previous history of DM were also categorised into the DM group, regardless of their admission blood glucose level. Patients with a blood glucose level ≥7.8 mmol/l and <11.1 mmol/l were categorised as having peri-infarct dysglycemia. Patients with an admission blood glucose <7.8 mmol/l were considered as having normoglycemia. All patients had 18-month follow-up for clinical MACE (Major Adverse Cardiovascular Events; defined as cardiovascular death, recurrent myocardial infarction, coronary revascularization or hospital admission for cardiovascular cause).

### CMR protocol

All patients underwent CMR during their index admission. A follow up CMR study, using the identical imaging protocol, was performed after a median of 11 months to assess LV remodeling. Patients were studied supine in a 1.5 Tesla scanner (Philips Medical Systems, Best, The Netherlands) equipped with 'Master' gradients (30 mT/m peak gradients; 150 mT/m/ms slew rate) and a 5-element cardiac phased array receiver coil. Cine imaging covering the whole heart in 10-12 parallel short axis slices was performed using a steady state free precession (SSFP) pulse sequence (echo time (TE) 1.4 msec; repetition time (TR) 2.8 msec; flip angle 55°, spatial resolution 2 × 2 × 7 mm, 18 phases per cardiac cycle). A cumulative dose of 0.2 mmol/kg of Gadolinium-DTPA (Dimeglumine gadopentetate, Magnevist, Schering AG, Germany) was administered using a power injector (Spectris MR injection system, Medrad, USA). LGE images were acquired ten to fifteen minutes after contrast injection with an inversion-recovery segmented k-space gradient-echo pulse sequence with a non-selective 180° prepulse (TE 3.8 msec; TR 7.5 msec; flip angle 15°, spatial resolution 1.8 × 1.8 × 10 mm). The inversion time (TI) was adjusted to null the signal intensity from normal myocardium. Ten to 12 short axis slices were obtained.

### CMR analysis

**Figure 1:**
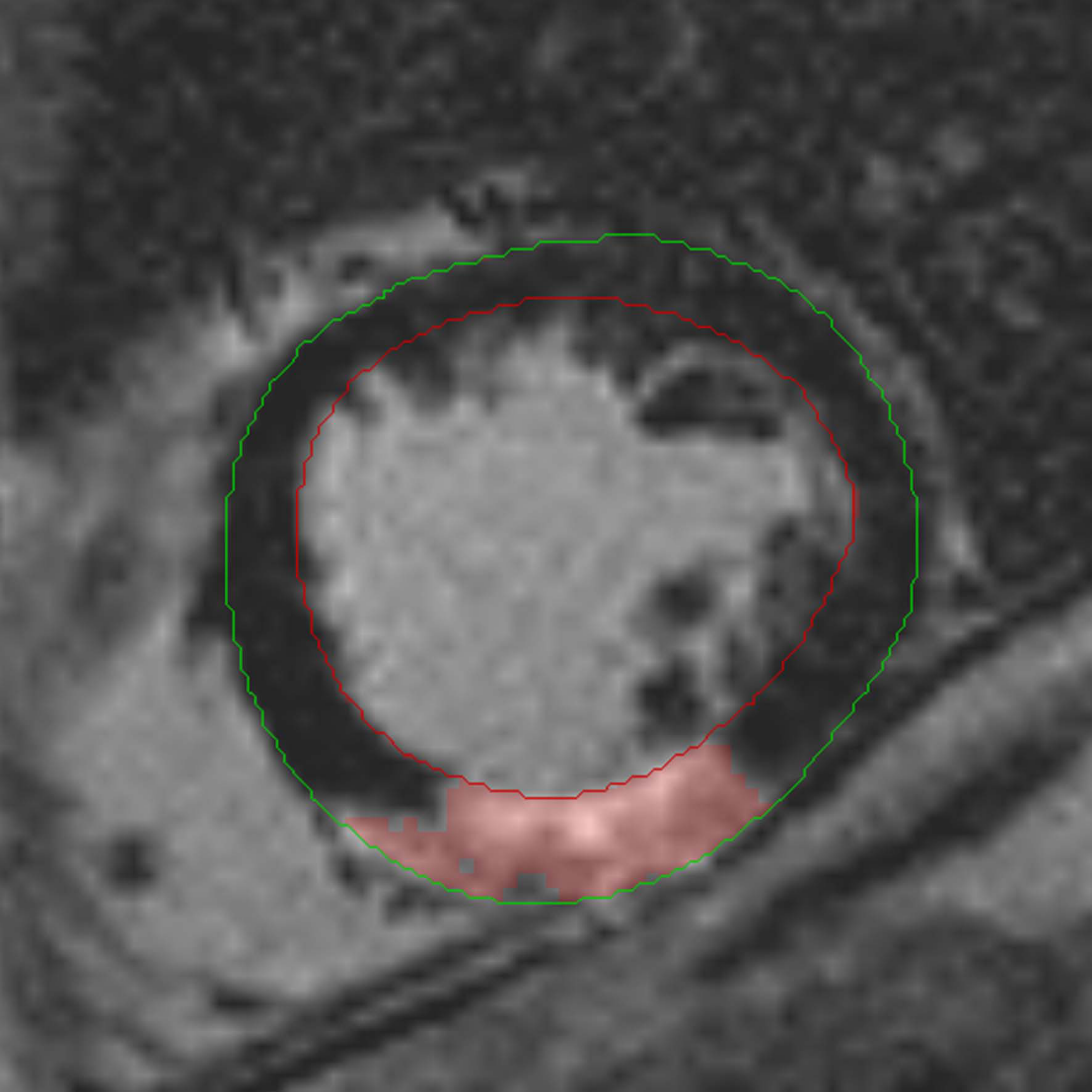
**An example of infarct contouring for quantification**. Late gadolinium enhanced short-axis slice through the left ventricle (LV). Green contour delineates epicardial LV border and red contour delineates endocardial LV border. Pink shaded area demarcates the area of late gadolinium hyperenhancement (i.e. signal intensity >2 SD of remote normal myocardium) which denotes myocardial infarct scar.

CMR analysis was performed off-line using commercial software (Mass 6.0, Medis, The Netherlands), by two experienced observers blinded to clinical details. In order to quantify LV mass and volumes, epicardial and endocardial contours were traced by manual planimetry on each short axis slice at end-diastole and end-systole. Using the summation of discs method, LV mass (g) was calculated from the total volume of myocardium at end-diastole multiplied by the myocardial density 1.05 g/ml. LGE images were displayed on a grey scale so as to optimally distinguish infarcted tissue (white) from normal myocardium (black) and the blood pool. Infarcted tissue was defined as areas with late gadolinium hyperenhancement. These regions were identified and then quantified using a semi-automated algorithm. Areas of hyperenhancement were defined as myocardium with a signal intensity >2 SD above the mean signal intensity of the remote normal myocardium [20] (Figure 1). The mass of infarcted myocardium was then automatically calculated. Transmural infarction was defined as the presence of any myocardial segment with >75% transmural extent of scar tissue on LGE images [16]. The presence of microvascular obstruction (MO) was also assessed in each patient. MO on LGE imaging was defined as a region of subendocardial hypoenhancement within the hyperenhanced region.

### Statistical analysis

The present study is an analysis of observational data so no formal power calculation to estimate sample size was performed prior to commencement. Statistical analysis was performed using SPSS, version 15.0, SPSS Inc, Chicago, USA. Two-sided p values ≤0.05 were considered to be statistically significant. Continuous CMR data are summarized as mean (standard deviation, SD) and categorical data as numbers (percentages). Continuous data between groups were compared using two sample t tests or one-way analysis of variance (ANOVA) tests with Bonferroni correction. Categorical data were analysed using Chi-squared tests. Event-free survival was calculated from the date of acute infarction to clinical outcome (MACE) occurrence. Patients who were lost to follow-up or did not experience an event were treated as censored. A log-rank test was used to compare the three groups (normoglycemia, dysglycemia, and diabetic), hazard ratios were estimated using a Cox proportional hazards model and Kaplan-Meier survival plots are presented.

## Results

Ninety-three patients completed baseline CMR at a median of 3 days (interquartile range (IQR), 3 to 4 days) from the index presentation. Seventy patients had follow-up CMR scans at a median of 328 days (IQR 191 to 382 days) from their index event. 13 patients declined the repeat scan, 4 patients relocated, 4 patients failed to attend and 2 patients died prior to follow up.

### Baseline characteristics

Twenty-two (24%) of the 93 patients were categorized into the DM group based on known clinical history of DM or the finding of a random blood glucose ≥11.1 mmol/L at the time of their hospital admission. 13/93 patients (14%) were categorized into the dysglycemia group on account of an admission blood glucose ≥7.8 mmol/l and <11.1 mmol/l. 58/93 patients (62%) were categorized into the normoglycemia group on the basis of an admission glucose <7.8 mmol/l. Clinical characteristics for patients categorized by glycemic status are provided in Table 1. There were no significant differences in terms of age, gender, cardiovascular risk factors, infarct location or Killip classification of heart failure between the three groups. Patients in the dysglycemia group and DM group had significantly lower total cholesterol levels than the normoglycemia group (mean (SD) 5.2 (1.04) vs. 4.7 (0.76) vs. 5.6 (0.9) mmol/l respectively; overall p < 0.001). These differences are most likely due to the higher incidence of pre-hospital use of statin therapy in the dysglycemia and DM groups. Of the 93 recruited patients, 71 suffered ST-elevation myocardial infarctions (STEMI) and were treated with thrombolysis, which was the standard therapy in our hospital at the time of study recruitment. The remaining patients (n = 22) had non-STEMI and were treated with optimal medical therapy followed by selective invasive coronary angiography if deemed high risk. Invasive coronary angiography and subsequent coronary revascularisation if appropriate, was performed if patients became clinically unstable or demonstrated evidence of myocardial ischemia either at rest or during supervised stress testing.

**Table 1:** Summary of baseline demographics and clinical characteristics.

		Normoglycemia N = 58	Dysglycemia N = 13	DM N = 22	P value
Age (years)		57 (10.7)	61 (11.1)	61 (10.8)	0.19
Male		83%	85%	91%	0.70
Hypertension		14%	15%	32%	0.22
Smoking		76%	69%	73%	0.55
Family History		40%	46%	45%	0.87
STEMI		69%	85%	91%	0.09
Territory of infarction	Anterior	43%	46%	45%	0.94
	Inferior	47%	54%	55%	
Killip class	I	81%	77%	64%	
	II	9%	0	18%	0.26
	III	10%	23%	18%	
Heart rate		73 (15.3)	78 (19)	81 (18.4)	0.14
Systolic BP (mmHg)		135 (25.6)	135 (17.4)	139 (23.4)	0.76
Diastolic BP (mmHg)		76 (17.3)	73 (15.7)	77 (15.9)	0.77
Peak CK (U/l)		1542 (1151)	2141 (1302)	2722 (1841)	0.003
Creatinine (μmol/l)		101 (13.6)	102 (11.7)	107 (17.7)	0.27
Cholesterol (mmol/l)		5.6 (0.9)	5.2 (1.0)	4.7 (0.76)	<0.001
Triglycerides (mmol/l)		1.8 (0.9)	2.1 (1.1)	1.6 (0.9)	0.40
Glucose (mmol/l)		6.3 (0.9)	9.1 (1.2)	12.5 (2.9)	<0.001

DM = diabetes mellitus, CK = creatine kinase, BP = blood pressure

Data represented as percentages or mean (SD).

### CMR characteristics

**Figure 2:**
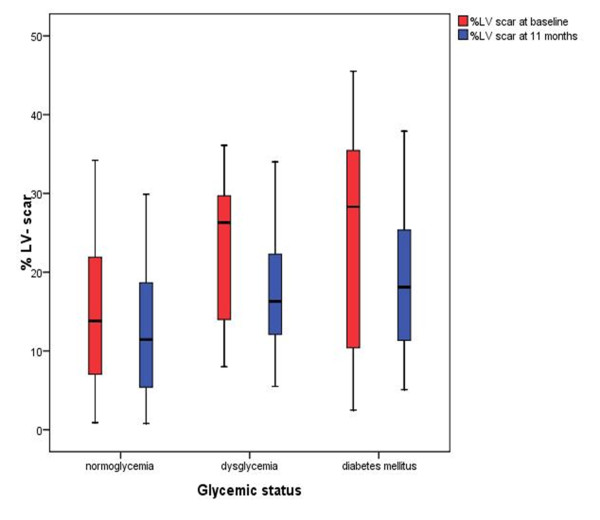
**Boxplot demonstrating% LV scar at baseline and at median follow up 11 months (n = 70) according to glycemic status**. The boxes represent the interquartile range and the lines denote the median. The error bars represent the 95% confidence intervals. (LV = left ventricular).

**Figure 3:**
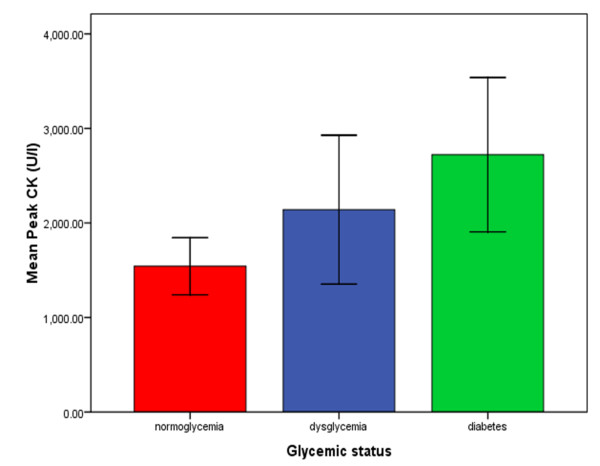
**Graph demonstrating the peak CK levels according to glycemic status**. The error bars represent the 95% confidence intervals. (CK = creatine kinase).

Patients with dysglycemia and DM had larger infarct sizes as determined by LGE-CMR, than those with normoglycemia, both at baseline (mean (SD)% LV scar 23.0 (10.9), 25.6 (12.9) and 15.8 (10.3) respectively; overall p = 0.001) and at 11 months after presentation (mean (SD)% LV scar 17.6 (8.9), 19.1 (9.6) and 12.4 (7.8) respectively; overall p = 0.017) (Figure 2). This was reflected in the peak CK measurements following AMI which were significantly higher in the dysglycemia and DM groups than the normoglycemia group (mean (SD) 2141 (1302), 2722 (1841) and 1542 (1151) U/l respectively; overall p = 0.003) (Figure 3). A greater proportion of patients with dysglycemia (12/13 (92%)) and diabetes mellitus (21/22 (95%)) had transmural infarctions (defined as >75% transmural extent of scar) when compared to patients with normoglycemia (44/58 (76%)). However, these difference were not significant (overall p = 0.09). Similarly, there were no significant differences in the proportions of patients with MO between the three groups (dysglycemia (6/13 (46%)) vs. diabetes (10/22 (45%)) vs. normoglycemia (19/58 (33%)), overall p = 0.10).

**Figure 4:**
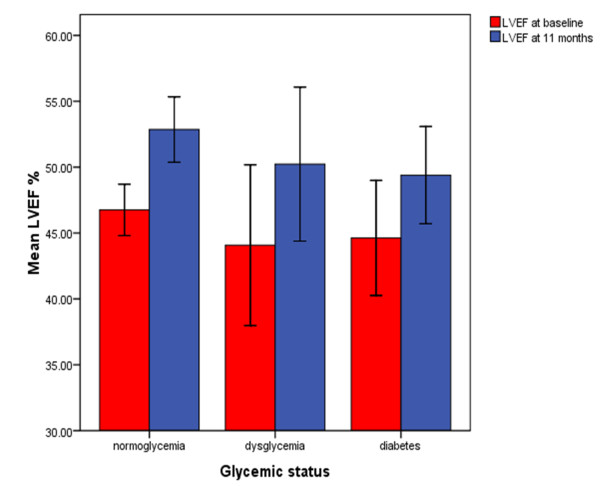
**Graph demonstrating mean LVEF% at baseline and at median follow up 11 months (n = 70) according to glycemic status**. The error bars represent the 95% confidence intervals. (LVEF = left ventricular ejection fraction).

There were no significant differences in LV ejection fraction (LVEF) between the three groups at baseline (mean (SD)% LVEF, dysglycemia 45 (8.5) vs. DM 43.4 (8.1) vs. normoglycemia 46.9 (7.1); overall p = 0.18) and at 11 months (mean (SD)% LVEF, dysglycemia 50.2 (7.6) vs. DM 49.4 (7.2) vs. normoglycemia 52.9 (8.2); overall p = 0.26) (Figure 4). The CMR results at baseline and at 11 months are summarized in Tables 2 and 3.

**Table 2:** CMR findings at baseline.

CMR parameters at baseline	Normoglycemia (n = 58)	Dysglycemia (n = 13)	DM (n = 22)	ANOVA P values
End diastolic volume (ml)	181 (37)	162 (37)	182 (25)	0.18
End systolic volume (ml)	97 (28)	91 (30)	103 (22)	0.39
Ejection fraction (%)	47 (7)	45 (8)	43 (8)	0.18
LV mass (g)	119 (29)	121 (31)	117 (19)	0.90
LV infarct size (%)	16 (10)	23 (11)	26 (13)	0.001

CMR = cardiovascular magnetic resonance, LV = left ventricular, other abbreviations as in Table 1.

Data represented as mean (SD).

**Table 3:** CMR findings in the 70 patients that had imaging performed at both baseline and at a median follow-up of 11 months.

	Normoglycemia (n = 44)	Dysglycemia (n = 9)	DM (n = 17)	ANOVA P- value
**LVEDV (ml)**				
Baseline	**178 (37)**	**159 (28)**	**179 (24)**	
Follow-up	**177 (35)**	**161 (40)**	**183 (36)**	**0.31**
P-value baseline vs. follow-up	**NS**	**NS**	**NS**	
**LVESV (ml)**				
Baseline	**95 (26)**	**89 (23)**	**99 (23)**	
Follow-up	**85 (27)**	**81 (30)**	**94 (29)**	**0.41**
P-value baseline vs. follow-up	**<0.001**	**NS**	**NS**	
**Ejection fraction (%)**				
Baseline	**47 (6)**	**44 (7)**	**45 (9)**	
Follow-up	**53 (8)**	**50 (7)**	**49 (7)**	**0.26**
P-value baseline vs. follow-up	**<0.001**	**<0.05**	**<0.05**	
**LV mass (g)**				
Baseline	**117 (29)**	**115 (21)**	**113 (18)**	
Follow-up	**99 (23)**	**95 (20)**	**102 (19)**	**0.74**
P-value baseline vs. follow-up	**<0.001**	**<0.001**	**<0.001**	
**%LV-scar**				
Baseline	**15 (9)**	**23 (11)**	**25 (14)**	
Follow-up	**12 (8)**	**18 (9)**	**19 (10)**	**0.02**
P-value baseline vs. follow-up	**<0.001**	**<0.05**	**<0.05**	

Data represented as mean (SD).

### Clinical cardiovascular outcome

**Figure 5:**
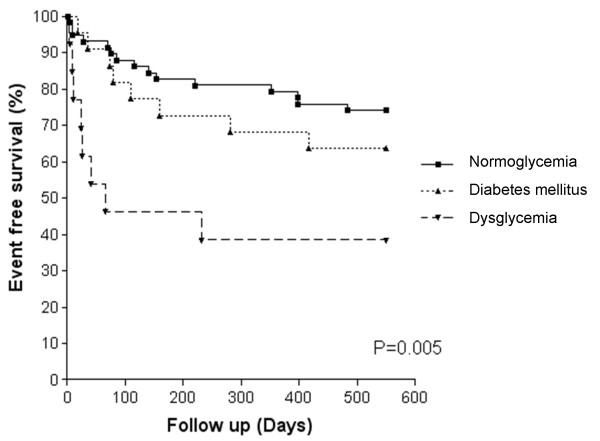
**Kaplan-Meier curves demonstrating event-free survival for all patients subdivided by glycemic status**.

Of the 58 patients with normoglycemia, 15 (25.9%) suffered at least one MACE, compared to 8 of the 13 patients (61.5%) with dysglycemia and 8 of the 22 patients (36.4%) in the DM group. From the entire cohort there were 3 deaths, 6 recurrent MIs, 11 revascularizations (9 PCI and 2 CABG), and 11 readmissions to hospital for a cardiovascular cause. There was a significant difference in overall event-free survival among the three groups of patients (log-rank test, p = 0.005) (Figure 5). Patients with dysglycemia were significantly more likely to experience an event at any time than normoglycemic patients, Hazard Ratio (HR) 3.82 (95% CI: 1.61, 9.06), but there was no significant difference in survival between patients with DM and normoglycemia, HR 1.48 (95% CI: 0.63, 3.50). When all patients with DM and peri-infarct dysglycemia were combined and compared to the normoglycemia group, as before 15/58 (25.9%) of those with normoglycemia suffered at least one MACE compared to 16/35 (45.7%) of those with DM and peri-infarct dysglycemia (χ2 = 3.87; p < 0.05).

## Discussion

In this study, we found that myocardial infarct size as measured by CMR is significantly larger in patients with dysglycemia or DM than in normoglycemic patients. This finding was supported by higher peak CK levels following AMI in the hyperglycemic subjects. Patients with dysglycemia and DM also had lower LV ejection fractions although these differences were not statistically significant. The differences in CMR findings between the three groups were consistent at 11 months follow-up.

Diabetes mellitus is firmly established as a strong independent risk factor for cardiovascular death and recurrent myocardial ischemia [21] and the prevalence of type 2 DM is predicted to rise over the next quarter of a century [22]. DM is also recognized as a surrogate marker for underlying cardiovascular disease. A recent Danish population-based study demonstrated that the excess cardiovascular risk associated with diabetes was not necessarily confined to middle-aged and older patients and that the very presence of medically-treated diabetes raised a diabetic individual's risk to that of a non-diabetic subject with a prior myocardial infarction [23].

Improved outcomes for normoglycemic patients suffering AMI over the last decade have not been matched by similar improvements in mortality for diabetic patients despite similar levels of baseline risk and appropriate medical therapy [24,25]. Patients with DM are known to have higher mortality in both the short and long term following AMI when compared with non-diabetic patients [26]. Furthermore, admission hyperglycemia appears to be an even stronger predictor of mortality in those patients without a prior diagnosis of DM [4,27]. Similarly in a study of AMI, amongst patients with no prior history of diabetes, a 1% absolute increase in HbA1c level at presentation resulted in a 24% increase in mortality [28].

The majority of studies that have investigated infarct size in diabetic patients have measured cardiac enzyme levels as the principal method of quantification. However, all of these studies, except one [29], have shown similar or smaller infarct size in diabetic patients. Enzymatic assessment of infarct size has its limitations, particularly in patients who have had reperfusion therapy [30]. It is recognised that successful reperfusion can increase the washout and hence the peak level of cardiac enzymes and therefore, paradoxically, lower peak enzyme levels may be associated with poorer reperfusion and possibly larger infarcts.

Alegria *et al*, used SPECT imaging following AMI in diabetic subjects and found significantly larger infarct size and reduced LVEF in patients with diabetes than non-diabetic patients [5]. One explanation given for this finding was that there was a higher incidence of previous MI in the diabetic population and that SPECT could not distinguish acute from chronic infarction. Our results are consistent with the previous data by Alegria, but our study population comprised only patients with a first episode of AMI and well-matched baseline clinical characteristics. There was no evidence, in any patient, of previous myocardial infarction in any other coronary artery territory on LGE imaging. By using the much more accurate technique of CMR to measure LV function and infarct size, we were able to demonstrate that patients with dysglycemia and DM had significantly larger infarcts which were associated with higher peak CK levels and a trend towards lower LVEF, when compared to normoglycemic patients. This is consistent with the relationship of CMR infarct size to the peak CK as shown by Younger *et al *[31]. The trend towards reduced LVEF in DM may be due to the larger infarct size or possibly other factors that were not recorded such as angiographic evidence of more extensive coronary artery disease or impaired myocardial perfusion. These results also support previous CMR data demonstrating a strong relationship between impaired glucose metabolism and myocardial damage in STEMI patients [32].

The 18-month outcome data from this study are particularly interesting and highlight an important message regarding risk stratification of AMI patients. We have shown that patients with an admission glucose level ≥7.8 mmol/l have a significantly reduced event-free survival from MACE. This is consistent with recent findings that in-hospital glucose levels are a stronger predictor of death than diabetes history and that patients with AMI with an admission glucose ≥8 mmol/l have a very high risk of death regardless of diabetes history [33]. Perhaps of greater interest is the fact that 18-month event free survival was actually lowest in those considered to have impaired glucose tolerance compared to those with DM or normoglycemia. Indeed, this group had smaller infarct sizes but worse outcome than those with DM. It may have been that those considered frankly diabetic had more aggressive secondary prevention and attention to glycemic control, whilst those with impaired glucose tolerance were actually under-diagnosed for DM. This would be in keeping with the findings from Norhammar *et al*, who showed in their series of AMI patients with random glucose levels below 11.1 mmol/L, that approximately one-third of patients had overt DM and a further third had impaired glucose tolerance at discharge and 3 months later [21]. Clearly infarct size and ventricular function/remodeling are important determinants of outcome, with larger infarcts expected to manifest as greater risk of sudden cardiac death (arrhythmia) and heart failure. However, other pathophysiological mechanisms of infarct healing may be important including residual ischemic burden (hibernation), microvascular obstruction and hemorrhage within the infarct core. The impact of glycemic control on these factors remains to be determined.

## Limitations

We made a pragmatic decision to categorize patients based on admission blood glucose levels. We acknowledge that more than one measurement of blood glucose is required to diagnose DM and impaired glucose tolerance but nevertheless our data highlight some important observations. The study population was also relatively small and a larger sample size would have allowed more detailed subgroup analyses. Data were not collected regarding on-going glycometabolic control or HbA1c levels and therefore we could not assess the significance of these variables on infarct size or event-free survival. The study population included patients with ST-elevation MI and non-ST-elevation MI. Again, a larger population would have enabled us to analyse these groups separately. Although primary percutaneous coronary intervention (PPCI) is now considered the optimum reperfusion strategy for the treatment of STEMI [34], intravenous thrombolysis remains the mainstay of therapy for the majority of patients in the United Kingdom and USA. Therefore, the findings from this study are directly relevant to current cardiological practice, although it is accepted that in the future PPCI will supercede thrombolysis and this may have a direct impact on myocardial infarct size.

## Conclusions

The results of this study suggest that following AMI, patients with dysglycemia and DM sustain significantly larger myocardial infarctions than normoglycemic patients. This finding may be a significant factor contributing to the known higher mortality seen in these patients. In addition, dysglycemia at the time of AMI has a significant adverse impact on cardiovascular outcome, an effect which appears more marked even compared to those patients with diabetes mellitus. Further studies are needed to explore the significance of glycemic control on infarct size and ventricular remodeling in order to improve our understanding of the mechanisms that account for the adverse outcome in patients with dysglycemia and AMI.

## Authors' contributions

The contributions of each author to this manuscript are as follows; ANM was involved in analysis and interpretation of the data, drafting of the manuscript and final approval of the manuscript. AC, NA, GW, SGB and SP were involved in the design of the study, analysis of the data, revising the manuscript and giving final approval. JPG was responsible for the conception and design of the study, analysis and interpretation of the data, drafting the manuscript and giving final approval of the paper.

## Declaration of competing interests

The authors declare that they have no competing interests.
